# Statistical evaluation of adding multiple risk factors improves Framingham stroke risk score

**DOI:** 10.1186/s12874-017-0330-8

**Published:** 2017-04-14

**Authors:** Xiao-Hua Zhou, Xiaonan Wang, Ashlee Duncan, Guizhou Hu, Jiayin Zheng

**Affiliations:** 1 Changchun University of Chinese Medicine Affiliated Hospital, Changchun, Jilin China; 2 Department of Biostatistics, School of Public Health, University of Washington, Seattle, WA 98195 USA; 3 School of Statistics, Renmin University of China, Beijing, 100872 China; 4 BioSignia, Inc, Durham, NC USA; 5 Department of Biostatistics and Bioinformatics, Duke University, Durham, USA; 6 School of Mathematical Sciences, Peking University, Beijing, China

**Keywords:** Framingham model, C-statistics, Synthesis analysis, NRI, Discrimination, Calibration, Reclassification

## Abstract

**Background:**

Framingham Stroke Risk Score (FSRS) is the most well-regarded risk appraisal tools for evaluating an individual’s absolute risk on stroke onset. However, several widely accepted risk factors for stroke were not included in the original Framingham model. This study proposed a new model which combines an existing risk models with new risk factors using synthesis analysis, and applied it to the longitudinal Atherosclerosis Risk in Communities (ARIC) data set.

**Methods:**

Risk factors in original prediction models and new risk factors in proposed model had been discussed. Three measures, like discrimination, calibration and reclassification, were used to evaluate the performance of the original Framingham model and new risk prediction model.

**Results:**

Modified C-statistics, Hosmer-Lemeshow Test and classless NRI, class NRI were the statistical indices which, respectively, denoted the performance of discrimination, calibration and reclassification for evaluating the newly developed risk prediction model on stroke onset. It showed that the NEW-STROKE (new stroke risk score prediction model) model had higher modified C-statistics, smaller Hosmer-Lemeshow chi-square values after recalibration than original FSRS model, and the classless NRI and class NRI of the NEW-STROKE model over the original FSRS model were all significantly positive in overall group.

**Conclusion:**

The NEW-STROKE integrated with seven literature-derived risk factors outperformed the original FSRS model in predicting the risk score of stroke. It illustrated that seven literature-derived risk factors contributed significantly to stroke risk prediction.

## Background

Despite a consistent decline in stroke mortality over the last twenty-plus years, cerebrovascular disease remains a major cause of death and disability in the U.S. and worldwide [1–4]. Epidemiological studies in various populations have reported numerous risk factors for stroke, including age, ethnicity, blood pressure, pre-existing cardiovascular disease, and diabetes mellitus [5–13]. Integrating these reported factors, several assessment models have been developed to evaluate an individual’s absolute risk for stroke onset [14–20]. One of the most well-regarded stroke risk appraisal tools is the Framingham Stroke Risk Score (FSRS), as developed from the Framingham Heart Study [14–16, 21]. However, despite it prominence, several widely accepted risk factors for stroke, such as body weight and shape [22–26] and family history [27–30] were not included in the original Framingham model. The objective of the present study was to evaluate whether the FSRS could be improved upon by developing a new stroke model that combines the original Framingham algorithm with seven additional literature-derived risk factors; this new model was created using a novel modeling process, called synthesis analysis [31, 32]. We used the third National Health and Nutrition Examination Survey (NHANES III) to develop our new model. Both the new model and the FSRS model were then evaluated objectively and scientifically using statistical measures commonly used to assess the performance of prediction models--- discrimination, calibration, and reclassification---in the longitudinal Atherosclerosis Risk in Communities (ARIC) dataset.

## Methods

### Stroke prediction models

In modern medicine, prediction models have become valuable and important tools for risk education, disease prevention, and treatment-strategy selection. Often considered the gold standard for risk prediction estimates, data from the Framingham cohort have been used to develop risk functions to evaluate several clinical outcomes, including coronary heart disease, atrial fibrillation, congestive heart failure, diabetes, and hypertension, among others [33–37].

In the original publication, stroke risk appraisal functions were developed among Framingham subjects who were 55 to 84 years of age, free of stroke at baseline, and followed for 10 years [15]. Risk factors included in the 1991 stroke profile functions included: age, sex, systolic blood pressure, use of antihypertensive medication, presence of diabetes mellitus, current cigarette smoker status, and the presence of pre-existing cardiovascular disease (coronary heart disease, cardiac failure, or intermittent claudication), atrial fibrillation, and/or left ventricular hypertrophy by electrocardiogram.

After publication, review of the cohort data indicated that the additive effect for antihypertensive therapy was applicable only when systolic blood pressure ranged from 110 to 200 mmHg, rather than across all levels of systolic blood pressure as originally reported. The algorithms were revised accordingly, and the modified risk appraisal function is the current Framingham Stroke Risk Score [16]. The detailed procedure of using Framingham Stroke Risk Score for predicting a future patient is given in Appendix 1.

Despite its widespread recognition, several generally accepted risk factors were not included in the original Framingham Stroke Risk Score (FSRS). To overcome this limitation, a novel model-building method, called synthesis analysis, was proposed to incorporate new risk factors into the existing FSRS model. Synthesis analysis is a statistical method used to develop new, comprehensive risk assessment models by combining literature-derived risk factors with an existing prediction model. The primary advantage of synthesis analysis is that it allows for the updating of an existing risk prediction model with new risk factors without needing to collect comprehensive information on all original and new risk factors and then following the cohort participants for outcomes of interest, which may take a long time. Detailed descriptions of synthesis analysis and its statistical validation have been reported elsewhere [31, 32, 38–40].

Adding to the baseline, multivariate FSRS equation, the new stroke model incorporates several literature-derived risk factors, including African American ethnicity, physical exercise level (low, moderate, or high based on frequency and intensity of activity), body mass index (kg/m2), waist circumference, height, HDL cholesterol, and use of hormone replacement therapy in postmenopausal females only. We will give a detailed procedure of constructing NEW-STROKE model using synthesis analysis method in Appendix 2.

### Measures of performance of a risk prediction model for survival time

Discrimination measures the ability of the model to distinguish subjects who will likely develop an event from those who will likely not. For a prediction model evaluating time to event (survival time), discrimination is typically measured by the concordance probability (denoted as C), which is defined as the probability of concordant pairs, where a pair of subjects is called concordant if the individual with the shorter survival time had the higher predicted risk.

Several approaches for calculating the concordance probability, also called C-statistics, have been proposed by Harrell [41, 42]. However, appropriate ordering of survival time in the presence of censoring is the key question when calculating C-statistics based on a rank-correlation measure by the Harrell method. Subsequently, a modified C-statistic was proposed by Uno [43], which consistently estimated a conventional concordance measure that is free of censoring. In this study, we utilized the modified C-statistics to evaluate the discrimination of the models.

Although discrimination measures the ability of a prediction model to distinguish a subject who will likely develop an event from a subject who will likely not develop an event, it does not measure the ability of a model to predict the probability of an event. Calibration describes how closely the predicted probabilities agree numerically with the actual outcomes [44]. If the new model is lack of fit in the ARIC data set, we need to recalibrate the model so that the overall predicted incidence equal to the overall observed incidence. The more detailed discussion on recalibration process is given in Appendix 3.

When comparing two predictive models, it is prudent to evaluate whether one model is better than another in classifying patients into clinically meaningful categories, such as high-, intermediate-, and low-risk groups. The net reclassification index (NRI) has been proposed to accomplish this task [45]. The NRI measures the change in clinically meaningful categories between two predictive models. In our study, the outputs of FSRS model and new model we developed are the estimated probability of having a stroke for each patient. There are two different definitions about NRI. One is called class NRI if we classify the estimated probability of having stroke into several levels, for example, three levels, like high-, intermediate- and low-risk groups. The other is classless NRI when we treat the estimated probability of having a stroke as continuous.

### Data set

The longitudinal Atherosclerosis Risk in Communities (ARIC) dataset was used for model evaluation and comparison. ARIC is a prospective epidemiologic study conducted in four US communities, and stroke incidence was recorded during the follow-up interval. Details of the ARIC study design have been previously described [46]. Briefly, stroke events were identified in a cohort of 15,792 individuals aged 45 to 64 years who were recruited from 1987 to 1989 among 4 communities in the United States: Washington County, Maryland; the northwest suburbs of Minneapolis, Minnesota; Forsyth County, North Carolina; and Jackson, Mississippi. Only African Americans were enrolled at the Jackson site; recruits from other sites were representative of their respective community. ARIC participants were reexamined every three years through 1998, and again in 2009. Health status follow-up occurs annually or semi-annually via telephone.

The ARIC study dataset, as used in the current research, contained 15,620 individuals, aged 45 to 64 years at baseline. To allow for appropriate comparisons between the models (FSRS and NEW-STROKE), all ARIC individuals with stroke at baseline (*n* = 172) were excluded from analysis. In the 10 years after baseline evaluation, 759 incident stroke cases, defined as clinically diagnosed stroke, receipt of stroke-related clinical procedures, or a fatal stroke event, were reported. Details of the identification and classification of stroke events in ARIC are described in greater detail elsewhere [47]. In addition, the visit 1 ARIC data did not contain the left ventricular hypertrophy by electrocardiogram FSRS input; therefore, all participants were assigned left ventricular hypertrophy = no.

## Results

As is shown in Table 1, the new model (NEW-STROKE) demonstrated a higher modified C-statistics than the Framingham model (FSRS) in the overall group and the female subgroup. However, for the male subgroup, the values of modified C-statistics between NEW-STROKE and FSRS are almost identical.

**Table 1:** Uno’s C-statistic

Uno’s C-statistic				
Overall	c.index	se	Lower95	Upper95
Framingham	0.762	0.207	0.739	0.779
NEW-STROKE	0.783	0.206	0.763	0.803
Male	c.index	se	Lower95	Upper95
Framingham	0.765	0.303	0.732	0.797
NEW-STROKE	0.765	0.287	0.733	0.790
Female	c.index	se	Lower95	Upper95
Framingham	0.769	0.317	0.737	0.800
NEW-STROKE	0.793	0.307	0.758	0.818

Hosmer-Lemeshow chi-square statistics are used to assess the calibration of a model by measuring the fitness between observed outcomes and predicted probabilities. Hosmer-Lemeshow chi-square test results are illustrated in Table 2. After recalibration, the test for NEW-STROKE is indicative of no lack of fit, since the test value is significantly smaller than the Hosmer-Lemeshow chi-square accepted threshold of 20.

**Table 2:** Hosmer-Lemeshow test

Hosmer-Lemeshow Test: 10 partition		
Overall	Framingham	NEW-STROKE
Before recalibration	12.1	43.2
After recalibration	12.4	4.7
Male	Framingham	NEW-STROKE
Before recalibration	17.0	26.3
After recalibration	17.8	5.8
Female	Framingham	NEW-STROKE
Before recalibration	12.3	22.2
After recalibration	12.1	8.2

Results for classless NRI are shown in Table 3. For the overall group, both before and after recalibration, the classless NRI values of the NEW-STROKE over Framingham are statistically significant. Following recalibration, the classless NRI are significantly positive in the non-event subgroup, while not significant in event subgroup. Before recalibration, all classless NRI values are significantly positive except for the female, non-event subgroup in which the classless NRI value is significantly negative.

**Table 3:** Classless NRI

Classless NRI				
Overall		point est	Lower95	Upper95
Before recalibration	overall	0.463	0.336	0.577
	event	0.444	0.312	0.559
	nonevent	0.020	0.003	0.036
After recalibration	overall	0.406	0.263	0.557
	event	−0.062	−0.206	0.088
	nonevent	0.468	0.453	0.481
Male		point est	Lower95	Upper95
Before recalibration	overall	0.469	0.290	0.635
	event	0.373	0.192	0.532
	nonevent	0.097	0.073	0.122
After recalibration	overall	0.436	0.217	0.632
	event	−0.102	−0.317	0.100
	nonevent	0.538	0.516	0.557
Female		point est	Lower95	Upper95
Before recalibration	overall	0.490	0.290	0.648
	event	0.534	0.342	0.695
	nonevent	−0.044	−0.068	−0.023
After recalibration	overall	0.451	0.245	0.663
	event	0.016	−0.192	0.229
	nonevent	0.435	0.415	0.454

For the class NRI, test data were categorized into three risk groups based on the quantiles of predicted risk for each model. Two types of thresholds were used: (1) the risk thresholds of lower than 70% quantile, 70 ~ 90% quantiles, and higher than 90% quantile; and (2) the risk thresholds of lower than 95% quantile, 95 ~ 99% quantiles, and higher than 99% quantile. The second threshold type was chosen because the estimated event probability is approximately 1% in the overall group and in both gender subgroups. The results are shown in Table 4. Evaluating the first threshold group, only the class NRI values in the overall and male non-event subgroups are significantly positive. For the second threshold type, all class NRI values are not statistically significant from 0.

**Table 4:** Class NRI

Class NRI			
0 ~ 0.7, 0.7 ~ 0.9, 0.9 ~ 1			
Overall	point est	lower95	Upper95
NRI	0.028	−0.056	0.115
Event NRI	0.016	−0.069	0.103
Nonevent NRI	0.012	0.004	0.020
Male	point est	lower95	Upper95
NRI	−0.027	−0.133	0.099
Event NRI	−0.040	−0.145	0.083
Nonevent NRI	0.013	0.002	0.025
Female	point est	lower95	Upper95
NRI	0.022	−0.117	0.152
Event NRI	0.011	−0.131	0.141
Nonevent NRI	0.011	−0.001	0.021
0 ~ 0.95, 0.95 ~ 0.99, 0.99 ~ 1			
Overall	point est	lower95	Upper95
NRI	0.053	−0.030	0.129
Event NRI	0.050	−0.032	0.126
Nonevent NRI	0.003	−0.001	0.006
Male	point est	lower95	Upper95
NRI	0.030	−0.087	0.148
Event NRI	0.027	−0.089	0.145
Nonevent NRI	0.003	−0.002	0.009
Female	point est	lower95	Upper95
NRI	0.056	−0.066	0.158
Event NRI	0.053	−0.065	0.156
Nonevent NRI	0.003	−0.003	0.007

Plots and charts were used to supplement the numeric comparisons between the NEW-STROKE and FSRS models and to provide additional information related to the detailed changes. A scatterplot of *p*_*new*_ versus *p*_*old*_ is a natural complement to the summary statistics already reported, with different symbols or separate plots for cases (events) and controls (non-events). A line at *p*_*new*_ = *p*_*old*_, in addition to horizontal and vertical lines at key risk thresholds, allows for the illustration of the extent and direction of change in risk induced by the NEW-STROKE model over the existing FSRS. A summary index, such as classless NRI or its components, classless event NRI, and classless non-event NRI, cannot distinguish between a few large upward movements, a medium number of small upward movements, or a large number of movements in both upward and downward directions. However, such important distinctions may be evident from a scatterplot.

**Fig. 1:**
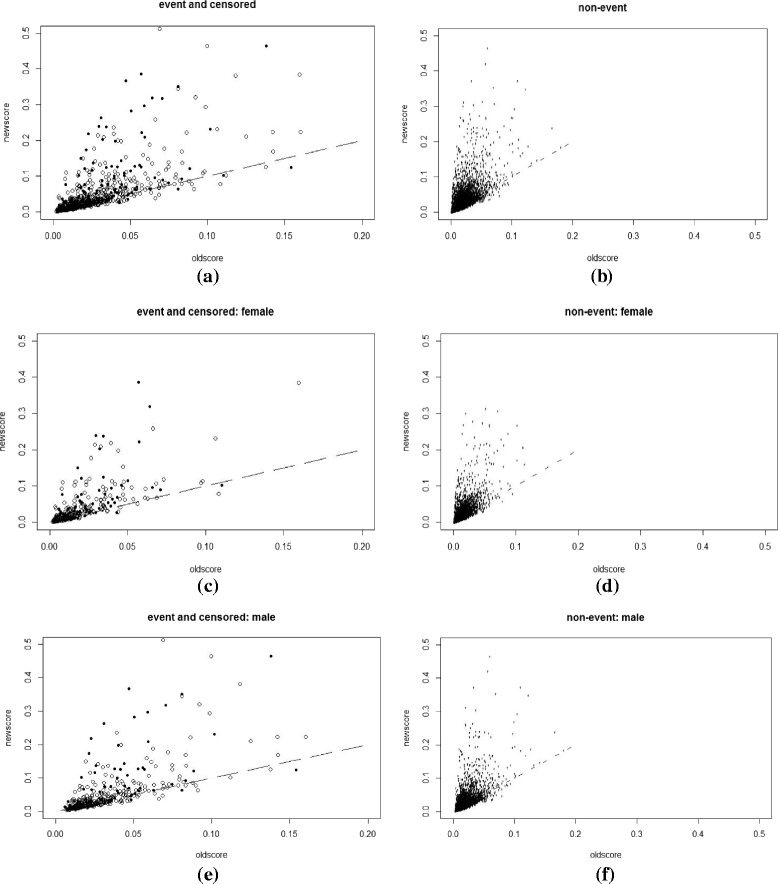
The scatterplot of *p*_*new*_ versus *p*_*old*_ **before** recalibration: separate plots for cases (definitely events and censored ones) and controls (definitely non-events) with the ranges of *p*_*new*_ and *p*_*old*_ being 0 ~ 0.5 and 0 ~ 0.2(or 0 ~ 0.5). **a** and **b** whole group, **c** and **d** female subgroup and **e** and **f** male subgroup

**Fig. 2:**
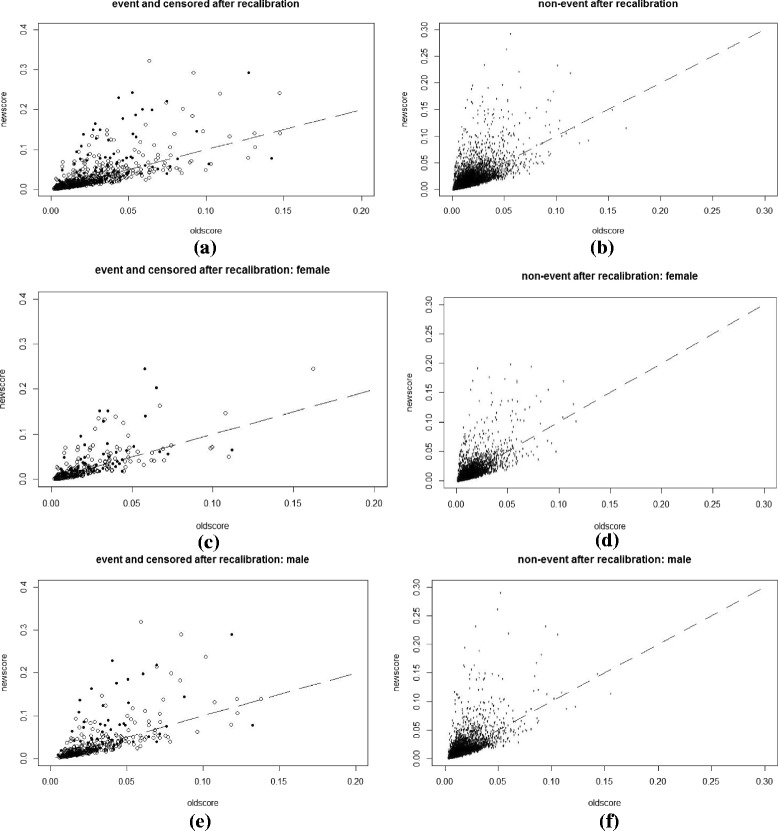
The scatterplot of *p*_*new*_ versus *p*_*old*_ **after** recalibration: separate plots for cases (definitely events and censored ones) and controls (definitely non-events) with the ranges of *p*_*new*_ and *p*_*old*_ being 0 ~ 0.4(or 0 ~ 0.3) and 0 ~ 0.2(or 0 ~ 0.3). **a** and **b** whole group, **c** and **d** female subgroup and **e** and **f** male subgroup

**Fig. 3:**
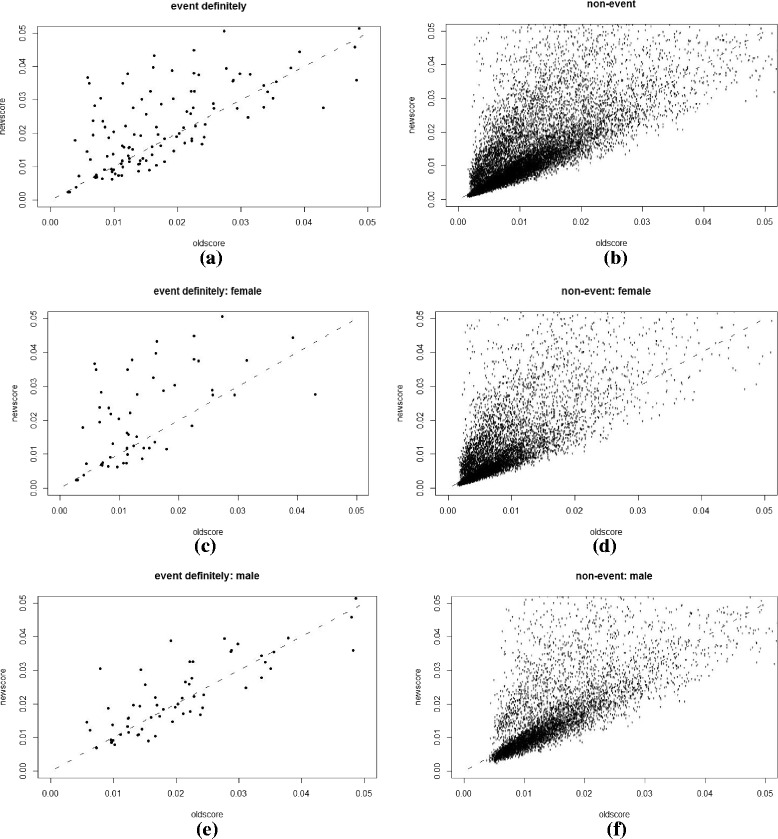
The scatterplot of *p*_*new*_ versus *p*_*old*_ **before** recalibration: separate plots for cases (definitely stroke events) and controls (definitely non-events) with the ranges of *p*_*new*_ and *p*_*old*_ being 0 ~ 0.05. **a** and **b** whole group, **c** and **d** female subgroup and **e** and **f** male subgroup

**Fig. 4:**
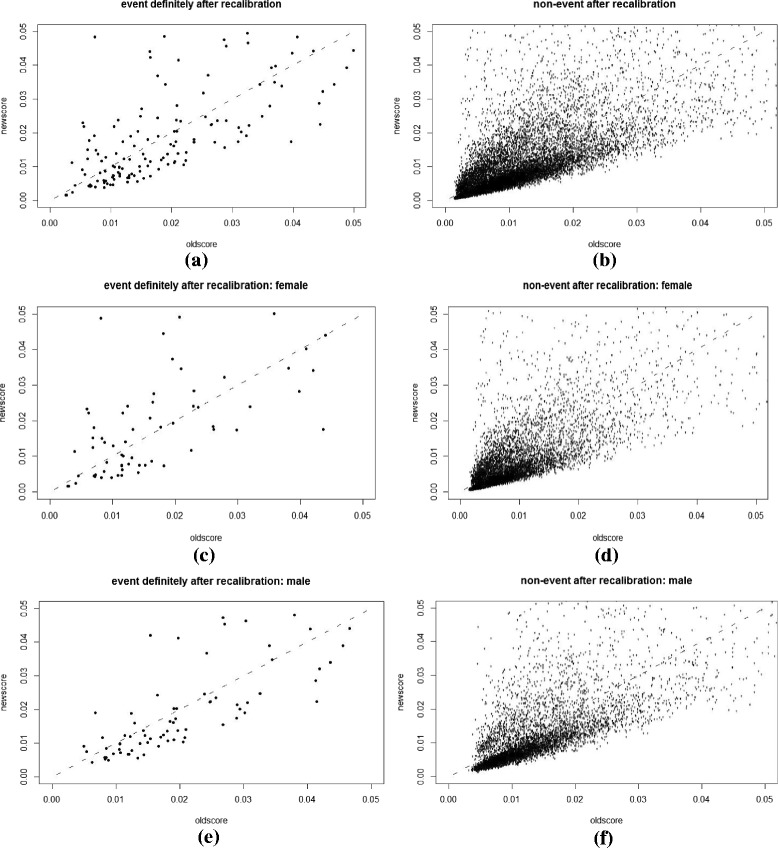
The scatterplot of *p*_*new*_ versus *p*_*old*_ **after** recalibration: separate plots for cases (definitely stroke events) and controls (definitely non-events) with the ranges of *p*_*new*_ and *p*_*old*_ being 0 ~ 0.05. **a** and **b** whole group, **c** and **d** female subgroup and **e** and **f** male subgroup

Scatterplots of *p*_*new*_ versus *p*_*old*_, with separate plots for cases (events) and controls (non-events) are shown in Figs. 1, 2, 3 and 4. They were plotted before and after recalibration, among the overall group, and also stratified by gender. Figures 3 and 4 are the enlarged main parts of Figs. 1 and 2, respectively.

## Discussion

In this study, a newly developed risk assessment model for stroke onset was shown by several statistical indicators to be superior to the gold-standard Framingham Stroke Risk Score. This new stroke risk score model(NEW-STROKE) was developed using a novel, model-building technology, called synthesis analysis, that allowed for the incorporation of seven additional literature-derived risk factors into the original FSRS.

Compared with the discrimination of these two models, the NEW-STROKE model had higher modified C-statistics than the original FSRS model in the overall group and in the female subgroup in the presence of censoring for survival time. This observation illustrates that the NEW-STROKE model has higher precision in both the overall group and the female subgroup in predicting stroke risk score. When evaluating calibration, the NEW-STROKE model outperformed the original FSRS model as evidenced by smaller Hosmer-Lemeshow chi-square values (<20) after recalibration in the overall group and in both gender subgroups. For reclassification, the NRIs represent the true- and false-positive rates, measuring the change between the NEW-STROKE model and the original FSRS model in predicting stroke risk score. The class NRI and the classless NRI of the NEW-STROKE model over the original FSRS model in the overall group were all significantly positive. This finding indicates that the NEW-STROKE model is better than the original FSRS model in correctly classifying individuals into higher- and lower-risk groups. Importantly, in the NEW-STROKE model, all seven literature-derived risk factors contributed significantly to stroke risk prediction, thereby effectively improving the original, baseline FSRS model.

This procedure of evaluating the NEW-STROKE model using statistical measures to assess prediction accuracy is similar to that used to evaluate a NEW-CHD model for coronary heart disease, the latter of which was shown to improve upon the gold-standard Framingham CHD Risk Score [38]. Numerous clinical publications report the development of new CHD risk prediction equations that are purportedly better than the original Framingham CHD score. Tzoulaki [48] disputed these claims of improvement citing notable methodological deficiencies in the majority of these publications, including incorrect validation study design (i.e., using the same data to develop and validate the model, improper selection of the study population, and/or incorrect definition of the outcome of interest), incomplete use of statistical evaluation criteria in model comparisons, and publication bias, among others. These deficiencies represent a prevalent issue in this type of research, and necessary steps should be taken to avoid them in similar, future studies. In the current research, we have successfully demonstrated that the NEW-STROKE model outperforms the original FSRS and have avoided Tzoulaki’s noted pitfalls [48], as follows.

First, to address the deficiency of incorrect validation study design, the NEW-STROKE model was developed using the peer-reviewed, clinical literature and the synthesis analysis method. It was not empirically derived within the ARIC data. It is notable that this validation study of the NEW-STROKE model, which does not have restrictions on the eligible population, evaluated only those ARIC participants without stroke at baseline; this exclusion allows for appropriate comparisons to the FSRS.

Second, in this research, the performance of the NEW-STROKE model was evaluated using three prediction accuracy properties--discrimination, calibration, and reclassification. These statistical indexes were calculated by appropriate approaches that were satisfied within the ARIC data. Furthermore, a modified C-statistic was used, as proposed by Uno [43], which specializes in accounting for censored survival data. These calculated accuracy properties were evaluated comprehensively to avoid another of Tzoulaki’s deficiencies in assessing a new prediction model.

To address the publication bias deficiency, the primary objective of this research was to evaluate whether a stroke risk assessment model, which improved upon a baseline Framingham Stroke Risk Score equation, could be developed using synthesis analysis. Inevitably, conflicting interests and publication bias play a small part in all validation studies; however, not every model developer will bias its validation. The main goal of this research was to demonstrate improvement of the original FSRS model, resulting in a more accurate risk prediction model for stroke. The current results illustrate that the NEW-STROKE model did indeed outperform the original FSRS model in predicting the risk score of stroke.

## Conclusion

In this paper, a new stroke risk score prediction model (NEW-STROKE) was developed using synthesis analysis, which allowed for the integration of seven, literature-derived risk factors into the original FSRS model. The NEW-STROKE model was then validated in the ARIC dataset. Three statistical measures were used to evaluate the performance of the NEW-STROKE model over the original FSRS model-discrimination, calibration, and reclassification. Validation results indicated that the original FSRS model was indeed improved upon by the NEW-STROKE model in predicting the risk of stroke. These findings provide another successful instance of utilizing synthesis analysis to improve upon a baseline, multivariate risk assessment mode.

## Appendix 1

### FSRS model for predicting a future patient

Stroke risk factors in the original FSRS model included 9 variables such as age, sex, systolic blood pressure, use of antihypertensive medication, presence of diabetes mellitus, current cigarette smoker status, the presence of pre-existing cardiovascular disease, atrial fibrillation, and left ventricular hypertrophy, denoted by *X*_1_, *X*_2_, *X*_3_, *X*_4_, *X*_5_, *X*_6_, *X*_7_, *X*_8_, *X*_9_ respectively. Logistic regression and Cox proportional hazards regression model had been employed in developing this FSRS model. Separate models were developed for each sex. From the published literatures [15], we got the predicted probability that this individual will develop a stroke within *t* years is *p* = 1 − (*S*(*t*))^*B*^,where *S*(*t*) denoted the estimated probability of surviving without a stroke for individuals whose risk factors are equal to the mean values of those observed in the data, *t* indicated the number of years. *B* = *e* ^*A*^, *A* had different formulas for each sex.

For sex = female,

$$ A={b}_1{X}_1+{b}_3{X}_3+\cdots +{b}_9{X}_9-\left({b}_1\overline{X_1}+{b}_3\overline{X_3}+\cdots +{b}_9\overline{X_9}\right) $$

with $\overline{X_i}, i=1,3,\dots, 9$ indicating the value of the mean for the *i* th covariate in one population.

For sex = male,

$$ A={c}_1{X}_1+{c}_3{X}_3+\dots +{c}_9{X}_9-\left({c}_1\overline{X_1}+{c}_3\overline{X_3}+\cdots +{c}_9\overline{X_9}\right) $$

As an example, consider a *x*_1_ -year old woman with a systolic blood pressure of *x*_3_ mm Hg who does not take antihypertensive medication (*x*_4_ = 0), is free of diabetes (*x*_5_ = 0),smokes (*x*_6_ = 1),does not have a previous cerebrovascular disease (*x*_7_ = 0),does not have a history of atrial fibrillation (*x*_8_ = 0), and does not left ventricular hypertrophy (*x*_9_ = 0). The predicted probability that the individual will develop a stroke within 10 years is:

$$ \begin{array}{l} p=1-{\left( S(10)\right)}^B\\ {}\kern1.12em =1-{\left( S(10)\right)}^{exp\left({b}_1{x}_1+{b}_3{x}_3+{b}_4\times 0+{b}_5\times 0+{b}_6\times 1+{b}_7\times 0+{b}_8\times 0+{b}_9\times 0-\left({b}_1\overline{X_1}+{b}_3\overline{X_3}+\cdots +{b}_9\overline{X_9}\right)\right)}\\ {}\kern1.12em =1-{\left( S(10)\right)}^{exp\left({b}_1{x}_1+{b}_3{x}_3+{b}_6-\left({b}_1\overline{X_1}+{b}_3\overline{X_3}+\cdots +{b}_9\overline{X_9}\right)\right)}\end{array} $$

The more detailed about the original Framingham Stroke Risk score model please see the Framingham study reference [15].

## Appendix 2

### Synthesis analysis and NEW-STROKE model

Synthesis analysis is a statistical method used to develop a multivariate regression model integrating the incomplete regression models and correlations among the independent variables of interest. In our study, we used Synthesis analysis method to propose the NEW-STROKE model, which included seven more risk factors than the FSRS model in predicting a future patient. Specificity, FSRS model is one of the most well-regards stroke risk appraisal tools, and can be regarded as a baseline equation in developing the NEW-STROKE model. Additional seven risk factors contained African American ethnicity, physical exercise level, body mass index, waist circumference, height, HDL cholesterol and the use of hormone replacement therapy derived from different longitudinal studies and comprehensive meta-analysis of the medical literatures. Let *Z*_1_, *Z*_2_, ⋯, *Z*_7_ denote these seven covariates respectively.

There are some assumptions in developing the NEW-STROKE model for predicting the stroke risk score by synthesis analysis method. The key assumption is that the input information, namely the associations of each risk factor with the stroke score and correlations among the multiple risk factors, are representative in the population. Another one is that these seven risk factors we chose in adding to our new model must be available in the training set which used to construct the model. In our study, the third National Health and Nutrition Examination Survey (NHANES III) has been used to propose the NEW-STROKE model for predicting stroke risk score. Because the NHANES III data is a representative sample of the US population and can be regarded as the only “superpopulation”. And these variables are all available both in NHANES III and ARIC data. The third one is the correlations between these risk factors and outcome of Stroke are exchangeable across different studies. The detailed procedure of constructing NEW-STROKE is as follows.

The dependent variable is the logit of the probability of stroke outcome, denoted by *Y*. Framingham Stroke Risk Score model can be regarded as the gold standard for stroke risk prediction, and be considered as a baseline equation in developing new model. The following steps can illustrate the detailed synthesis analysis methodology.

Assume the baseline empirical logistic model is:

$$ L Y=\alpha +{\beta}_1{X}_1+{\beta}_2{X}_2+\cdots +{\beta}_9{X}_9 $$

with *LY* denoting the logit of *Y*.

The parameters *α*, *β*_1_, *β*_2_, ⋯, *β*_9_ had been estimated in Framingham Heart Study. We use $\widehat{\alpha}+\widehat{\beta_1}{X}_1+\widehat{\beta_2}{X}_2+\cdots +\widehat{\beta_9}{X}_9$ to predict *LY* for each patient who was followed up in the NHANES III. Denote the prediction by $\widehat{LY}$. The correlations between the additional seven risk factors (*Z*_1_, *Z*_2_, ⋯, *Z*_7_) and stroke outcome represent by *γ*_1_, *γ*_2_, ⋯, *γ*_7_, which derived from the medical literatures.

Firstly, we use $\widehat{\alpha}+\widehat{\beta_1}{X}_1+\widehat{\beta_2}{X}_2+\cdots +\widehat{\beta_9}{X}_9$ to calculate the predicted dependent variable $\widehat{LY}$ for each patient in the NHANES III.

Secondly, for the same dataset, $\widehat{LY}$ can be used as dependent variable, the first additional risk factor *Z*_1_ is the independent variable, build the linear regression model $\widehat{LY}={\delta}_1+{\varsigma}_1{Z}_1$. We use a weighted least square method to obtain the regression coefficient $\widehat{\varsigma_1}$. $\widehat{\varsigma_1}$ represents the association of $\widehat{\alpha}+\widehat{\beta_1}{X}_1+\widehat{\beta_2}{X}_2+\cdots +\widehat{\beta_9}{X}_9$ with *Z*_1_, also means that how much of *Z*_1_ is captured in the baseline equation. *γ*_1_ denotes the correlation between the first risk factor *Z*_1_ and stroke outcome.

Thirdly, we use the difference between *γ*_1_ and ${\widehat{\varsigma}}_1$ to reflect the association of the first risk factor *Z*_1_ with the stroke that was not captured in the baseline equation. The new equation has the form:

$$ L Y1=\widehat{LY}+\left({\gamma}_1-\widehat{\varsigma_1}\right){Z}_1=\widehat{\alpha}+\widehat{\beta_1}{X}_1+\widehat{\beta_2}{X}_2+\cdots +\widehat{\beta_9}{X}_9+\left({\gamma}_1-\widehat{\varsigma_1}\right)\left({Z}_1-\overline{Z_1}\right) $$

here, $\overline{Z_1}$ indicate the mean of *Z*_1_. Constant $\widehat{\alpha}$ keeps the same when *Z*_1_ is mean centered. Then, this new equation has been regarded as a new baseline equation. We continue to add the second interested risk factor to this new baseline equation. We repeat the first step to third step until all interested risk factors are included in the final model.

The final equation is:

$$ \begin{array}{l} LY7=\widehat{\alpha}+\widehat{\beta_1}{X}_1+\widehat{\beta_2}{X}_2+\cdots +\widehat{\beta_9}{X}_9+\left({\gamma}_1-\widehat{\varsigma_1}\right)\left({Z}_1-\overline{Z_1}\right)\\ {}\kern2em +\left({\gamma}_2-\widehat{\varsigma_2}\right)\left({Z}_2-\overline{Z_2}\right)+\cdots +\left({\gamma}_7-\widehat{\varsigma_7}\right)\left({Z}_7-\overline{Z_7}\right)\end{array} $$

*LY*7 is our proposed NEW-STROKE model by synthesis analysis method. The detailed steps are our procedure of synthesis analysis.

Therefore, the predicted probability of a patient who will develop a stroke is:$p=\frac{1}{1+ exp\left(- LY7\right)}$. We can use this formula to predict the probability of having a stroke for a future patient.

## Appendix 3

### Recalibration approach

The disease outcome *Y* = 1 if patient who had a stroke, otherwise, *Y* = 0.

The NEW-STROKE prediction model has the following form:

$$ \begin{array}{l} logit\frac{P\left( Y=1\Big| X\right)}{1- P\left( Y=1\Big| X\right)}=\widehat{\alpha}+\widehat{\beta_1}{X}_1+\widehat{\beta_2}{X}_2+\cdots +\widehat{\beta_9}{X}_9\\ {}\kern8.5em +\left({\gamma}_1-\widehat{\varsigma_1}\right)\left({Z}_1-\overline{Z_1}\right)+\left({\gamma}_2-\widehat{\varsigma_2}\right)\left({Z}_2-\overline{Z_2}\right)+\cdots +\left({\gamma}_7-\widehat{\varsigma_7}\right)\left({Z}_7-\overline{Z_7}\right)\end{array} $$

Therefore,

$$ \widehat{P}\left( Y=1\Big| X\right)=\frac{exp\left(\widehat{\alpha}+\widehat{\beta_1}{X}_1+\cdots +\widehat{\beta_9}{X}_9+\left({\gamma}_1-\widehat{\varsigma_1}\right)\left({Z}_1-\overline{Z_1}\right)+\cdots +\left({\gamma}_7-\widehat{\varsigma_7}\right)\left({Z}_7-\overline{Z_7}\right)\right)}{1+ exp\left(\widehat{\alpha}+\widehat{\beta_1}{X}_1+\cdots +\widehat{\beta_9}{X}_9+\left({\gamma}_1-\widehat{\varsigma_1}\right)\left({Z}_1-\overline{Z_1}\right)+\cdots +\left({\gamma}_7-\widehat{\varsigma_7}\right)\left({Z}_7-\overline{Z_7}\right)\right)} $$

Let the observed overall incidence *P*(*Y* = 1|*X*) = *ρ*. If the predicted probability $\widehat{P}\left( Y=1\Big| X\right)$ does not equal to the observed overall incidence *ρ*. We need to adjust this NEW-STROKE model, such as adjust the constant term $\widehat{\alpha}$. For example,

$$ \widehat{P}\left( Y=1\Big| X\right)=\frac{exp\left(\widehat{\alpha}+\widehat{\beta_1}{X}_1+\cdots +\widehat{\beta_9}{X}_9+\left({\gamma}_1-\widehat{\varsigma_1}\right)\left({Z}_1-\overline{Z_1}\right)+\cdots +\left({\gamma}_7-\widehat{\varsigma_7}\right)\left({Z}_7-\overline{Z_7}\right)+ C\right)}{1+ exp\left(\widehat{\alpha}+\widehat{\beta_1}{X}_1+\cdots +\widehat{\beta_9}{X}_9+\left({\gamma}_1-\widehat{\varsigma_1}\right)\left({Z}_1-\overline{Z_1}\right)+\cdots +\left({\gamma}_7-\widehat{\varsigma_7}\right)\left({Z}_7-\overline{Z_7}\right)+ C\right)} $$

so that $$ \widehat{P}\left( Y=1\Big| X\right)= P\left( Y=1\Big| X\right)=\rho $$.

After calculating, we are able to give the formula of *C*:

$$ \begin{array}{l} C= log\left(\frac{\rho}{1-\rho}\right)-\left\{\widehat{\alpha}+\widehat{\beta_1}{X}_1+\widehat{\beta_2}{X}_2+\cdots +\widehat{\beta_9}{X}_9\right.\\ {}\left.+\left({\gamma}_1-\widehat{\varsigma_1}\right)\left({Z}_1-\overline{Z_1}\right)+\left({\gamma}_2-\widehat{\varsigma_2}\right)\left({Z}_2-\overline{Z_2}\right)+\cdots +\left({\gamma}_7-\widehat{\varsigma_7}\right)\left({Z}_7-\overline{Z_7}\right)\right\}\end{array} $$

## Abbreviations

ARIC: Atherosclerosis risk in communities
FSRS: Framingham stroke risk score
NEW-STROKE: New stroke risk score model
